# 1-(Bromo­meth­yl)adamantane

**DOI:** 10.1107/S1600536811023695

**Published:** 2011-06-25

**Authors:** Jarmila Černochová, Andrea Čablová, Michal Rouchal, Marek Nečas, Robert Vícha

**Affiliations:** a Department of Chemistry, Faculty of Technology, Tomas Bata University in Zlin, Nám. T. G. Masaryka 275, Zlín,762 72, Czech Republic; b Department of Chemistry, Faculty of Science, Masaryk University, Kamenice 5, Brno-Bohunice, 625 00, Czech Republic

## Abstract

The title compound, C_11_H_17_Br, has crystallographically imposed mirror symmetry in the solid state with mol­ecules bis­ected by mirror planes parallel to the crystallographic *ac* plane (five C atoms, three H atoms and the Br atom lie on the mirror plane). The asymmetric unit contains one half-mol­ecule. The crystal packing is stabilized only *via* weak non-specific van der Waals inter­actions.

## Related literature

For the synthetic procedure, see: Nordlander *et al.* (1966 ▶). For the structure of a related non-polar adamantane derivate, see: Rouchal *et al.* (2010 ▶).
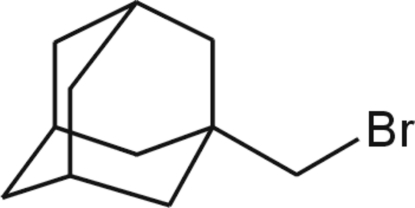


## Experimental

### 

#### Crystal data

C_11_H_17_Br
*M* *_r_* = 229.16
Monoclinic, 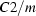

*a* = 10.7250 (3) Å
*b* = 7.0066 (3) Å
*c* = 13.4479 (4) Å
β = 101.801 (3)°
*V* = 989.19 (6) Å^3^
*Z* = 4
Mo *K*α radiation
μ = 4.10 mm^−1^
*T* = 120 K
0.40 × 0.40 × 0.30 mm

#### Data collection

Oxford Diffraction Xcalibur Sapphire2 diffractometer
Absorption correction: multi-scan (*CrysAlis RED*; Oxford Diffraction, 2009 ▶) *T*_min_ = 0.480, *T*_max_ = 1.000
5102 measured reflections
951 independent reflections
900 reflections with *I* > 2σ(*I*)
*R*_int_ = 0.014

#### Refinement

*R*[*F* ^2^ > 2σ(*F* ^2^)] = 0.017
*wR*(*F* ^2^) = 0.048
*S* = 1.08
951 reflections
64 parameters
H-atom parameters constrained
Δρ_max_ = 0.28 e Å^−3^
Δρ_min_ = −0.29 e Å^−3^

### 

Data collection: *CrysAlis CCD* (Oxford Diffraction, 2009 ▶); cell refinement: *CrysAlis RED* (Oxford Diffraction, 2009 ▶); data reduction: *CrysAlis RED*; program(s) used to solve structure: *SHELXS97* (Sheldrick, 2008 ▶); program(s) used to refine structure: *SHELXL97* (Sheldrick, 2008 ▶); molecular graphics: *ORTEP-3* (Farrugia, 1997 ▶) and *Mercury* (Macrae *et al.*, 2008 ▶); software used to prepare material for publication: *SHELXL97*.

## Supplementary Material

Crystal structure: contains datablock(s) global, I. DOI: 10.1107/S1600536811023695/jh2298sup1.cif

Structure factors: contains datablock(s) I. DOI: 10.1107/S1600536811023695/jh2298Isup2.hkl

Supplementary material file. DOI: 10.1107/S1600536811023695/jh2298Isup3.cml

Additional supplementary materials:  crystallographic information; 3D view; checkCIF report

## Figures and Tables

**Fig. 1.**
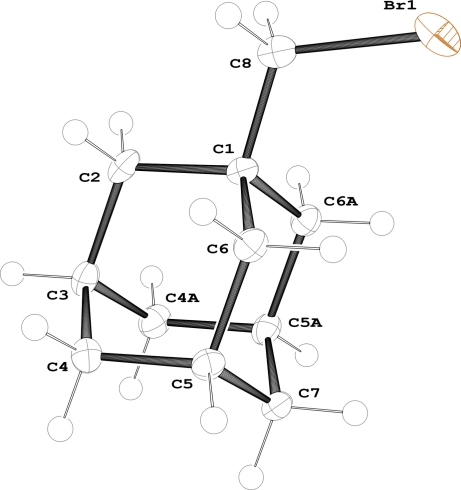
Molecular structure of of the title compound with atoms represented as 50% probability ellipsoids. Symmetry code used to generate the complete molecule: x, -y, z.

**Fig. 2.**
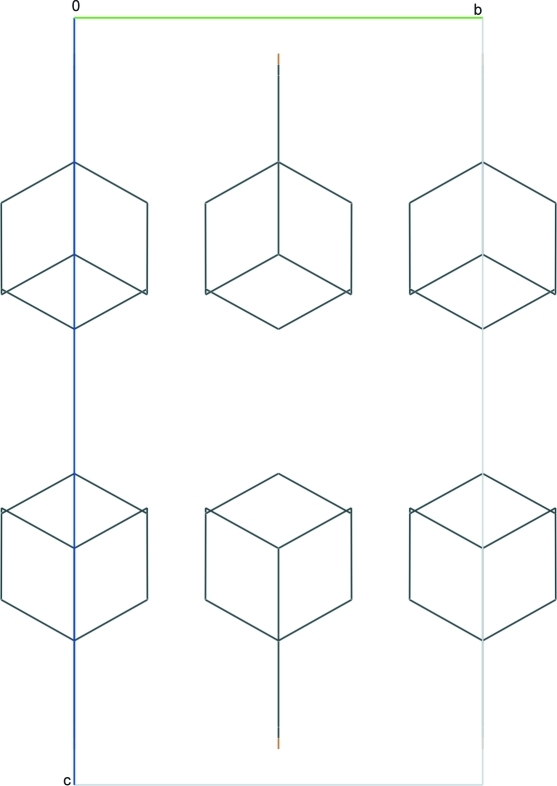
The unit cell viewed along the a-axis. H-atoms have been omitted for clarity.

## supplementary crystallographic information

### Comment

The title compound is well known and widely used in chemistry of adamantane derivates as convenient source of 1-adamantylmethyl substituent (Nordlander *et al.*, 1966). Although this compound is very easy to purify *via* crystallization or sublimation, it is prone to form soft thin plates. Due to this fact hand in hand with relatively low melting point (314–316 K), the crystal structure was not published yet. We successfully prepared sufficiently thick plates usable for XRD analyses *via* very slow partial evaporation of solvents from the solution of the title compound in DMF, petroleum ether, and ethyl acetate at room temperature. The molecule of the title compound contains the adamantane moiety consisting from three fused cyclohexane rings in classical chair conformation. The value of C—C—C angles varies within the range of 108.35 (6)–110.27 (12)°. No specific interactions, in addition to the van der Waals interactions, were observed to stabilize the packing of the molecules in the crystal.

### Experimental

The title compound was prepared according to slightly modified previously published procedure (Nordlander *et al.*, 1966). The mixture of starting 1-adamantylmethanol (23.3 g, 0.14 mol), ZnBr_2_ (80.7 g, 0.36 mol), and azeotropic hydrobromic acid (412 cm^3^) was refluxed until the GC analyses showed complete disappearing of starting alcohol. Mixture was extracted with hexane:diethyl ether (1:1, v:v), collected organic portions were successively washed with 10% sodium bicarbonate solution and brine and dried over Na_2_SO_4_. The evaporation of solvent yielded of 26.5 g (83%) of pale yellow soft plates.

### Crystal data

**Table:**

C_11_H_17_Br	*F*(000) = 472
*M**_r_* = 229.16	*D*_x_ = 1.539 Mg m^−^^3^
Monoclinic, *C*2/*m*	Melting point: 315 K
Hall symbol: -C 2y	Mo *K*α radiation, λ = 0.71073 Å
*a* = 10.7250 (3) Å	Cell parameters from 4311 reflections
*b* = 7.0066 (3) Å	θ = 3.1–27.2°
*c* = 13.4479 (4) Å	µ = 4.10 mm^−^^1^
β = 101.801 (3)°	*T* = 120 K
*V* = 989.19 (6) Å^3^	Block, colourless
*Z* = 4	0.40 × 0.40 × 0.30 mm

### Data collection

**Table:**

Oxford Diffraction Xcalibur Sapphire2 diffractometer	951 independent reflections
Radiation source: Enhance (Mo) X-ray Source	900 reflections with *I* > 2σ(*I*)
graphite	*R*_int_ = 0.014
Detector resolution: 8.4353 pixels mm^-1^	θ_max_ = 25.0°, θ_min_ = 3.1°
ω scans	*h* = −12→12
Absorption correction: multi-scan (*CrysAlis RED*; Oxford Diffraction, 2009)	*k* = −8→4
*T*_min_ = 0.480, *T*_max_ = 1.000	*l* = −15→15
5102 measured reflections	

### Refinement

**Table:**

Refinement on *F*^2^	Primary atom site location: structure-invariant direct methods
Least-squares matrix: full	Secondary atom site location: difference Fourier map
*R*[*F*^2^ > 2σ(*F*^2^)] = 0.017	Hydrogen site location: inferred from neighbouring sites
*wR*(*F*^2^) = 0.048	H-atom parameters constrained
*S* = 1.08	*w* = 1/[σ^2^(*F*_o_^2^) + (0.029*P*)^2^ + 0.8238*P*] where *P* = (*F*_o_^2^ + 2*F*_c_^2^)/3
951 reflections	(Δ/σ)_max_ < 0.001
64 parameters	Δρ_max_ = 0.28 e Å^−^^3^
0 restraints	Δρ_min_ = −0.29 e Å^−^^3^

### Special details

**Table:**

Geometry. All e.s.d.'s (except the e.s.d. in the dihedral angle between two l.s. planes) are estimated using the full covariance matrix. The cell e.s.d.'s are taken into account individually in the estimation of e.s.d.'s in distances, angles and torsion angles; correlations between e.s.d.'s in cell parameters are only used when they are defined by crystal symmetry. An approximate (isotropic) treatment of cell e.s.d.'s is used for estimating e.s.d.'s involving l.s. planes.
Refinement. Refinement of *F*^2^ against ALL reflections. The weighted *R*-factor *wR* and goodness of fit *S* are based on *F*^2^, conventional *R*-factors *R* are based on *F*, with *F* set to zero for negative *F*^2^. The threshold expression of *F*^2^ > 2σ(*F*^2^) is used only for calculating *R*-factors(gt) *etc*. and is not relevant to the choice of reflections for refinement. *R*-factors based on *F*^2^ are statistically about twice as large as those based on *F*, and *R*- factors based on ALL data will be even larger.

### Fractional atomic coordinates and isotropic or equivalent isotropic displacement parameters (Å^2^)

**Table:**

	*x*	*y*	*z*	*U*_iso_*/*U*_eq_	Occ. (<1)
Br1	0.35255 (2)	0.0000	0.046661 (18)	0.02707 (11)	
C1	0.1776 (2)	0.0000	0.18798 (16)	0.0146 (5)	
C2	0.0362 (2)	0.0000	0.19634 (17)	0.0175 (5)	
H2A	−0.0068	0.1144	0.1621	0.021*	0.50
H2B	−0.0068	−0.1144	0.1621	0.021*	0.50
C3	0.0259 (2)	0.0000	0.30840 (17)	0.0188 (5)	
H3	−0.0660	0.0000	0.3130	0.023*	
C4	0.09066 (14)	0.1784 (3)	0.36097 (12)	0.0199 (4)	
H4A	0.0834	0.1794	0.4332	0.024*	
H4B	0.0483	0.2943	0.3279	0.024*	
C5	0.23165 (14)	0.1783 (3)	0.35368 (12)	0.0169 (4)	
H5	0.2742	0.2948	0.3878	0.020*	
C6	0.24109 (14)	0.1789 (2)	0.24137 (12)	0.0163 (3)	
H6A	0.1988	0.2942	0.2077	0.020*	
H6B	0.3318	0.1824	0.2360	0.020*	
C7	0.2966 (2)	0.0000	0.40589 (17)	0.0183 (5)	
H7A	0.3878	0.0000	0.4021	0.022*	
H7B	0.2908	0.0000	0.4784	0.022*	
C8	0.1790 (2)	0.0000	0.07521 (18)	0.0202 (5)	
H8A	0.1329	0.1141	0.0435	0.024*	0.50
H8B	0.1329	−0.1141	0.0435	0.024*	0.50

### Atomic displacement parameters (Å^2^)

**Table:**

	*U*^11^	*U*^22^	*U*^33^	*U*^12^	*U*^13^	*U*^23^
Br1	0.03772 (17)	0.02270 (17)	0.02531 (16)	0.000	0.01703 (11)	0.000
C1	0.0154 (10)	0.0148 (12)	0.0125 (11)	0.000	0.0004 (8)	0.000
C2	0.0128 (10)	0.0162 (12)	0.0204 (12)	0.000	−0.0037 (9)	0.000
C3	0.0106 (10)	0.0236 (13)	0.0214 (12)	0.000	0.0015 (9)	0.000
C4	0.0163 (7)	0.0228 (10)	0.0203 (8)	0.0031 (7)	0.0034 (6)	−0.0032 (7)
C5	0.0148 (7)	0.0176 (9)	0.0173 (8)	−0.0026 (7)	0.0010 (6)	−0.0046 (7)
C6	0.0157 (7)	0.0145 (8)	0.0178 (8)	−0.0012 (7)	0.0014 (6)	−0.0001 (7)
C7	0.0132 (10)	0.0273 (14)	0.0135 (11)	0.000	0.0004 (8)	0.000
C8	0.0230 (11)	0.0195 (13)	0.0167 (11)	0.000	0.0010 (9)	0.000

### Geometric parameters (Å, °)

**Table:**

Br1—C8	1.975 (2)	C4—H4A	0.9900
C1—C8	1.520 (3)	C4—H4B	0.9900
C1—C6	1.533 (2)	C5—C7	1.529 (2)
C1—C6^i^	1.533 (2)	C5—C6	1.534 (2)
C1—C2	1.544 (3)	C5—H5	1.0000
C2—C3	1.533 (3)	C6—H6A	0.9900
C2—H2A	0.9900	C6—H6B	0.9900
C2—H2B	0.9900	C7—C5^i^	1.529 (2)
C3—C4	1.531 (2)	C7—H7A	0.9900
C3—C4^i^	1.531 (2)	C7—H7B	0.9900
C3—H3	1.0000	C8—H8A	0.9900
C4—C5	1.535 (2)	C8—H8B	0.9900
			
C8—C1—C6	111.91 (12)	C7—C5—C6	109.78 (14)
C8—C1—C6^i^	111.91 (12)	C7—C5—C4	109.45 (14)
C6—C1—C6^i^	109.67 (17)	C6—C5—C4	109.09 (12)
C8—C1—C2	106.47 (17)	C7—C5—H5	109.5
C6—C1—C2	108.36 (12)	C6—C5—H5	109.5
C6^i^—C1—C2	108.36 (12)	C4—C5—H5	109.5
C3—C2—C1	109.91 (17)	C1—C6—C5	110.28 (14)
C3—C2—H2A	109.7	C1—C6—H6A	109.6
C1—C2—H2A	109.7	C5—C6—H6A	109.6
C3—C2—H2B	109.7	C1—C6—H6B	109.6
C1—C2—H2B	109.7	C5—C6—H6B	109.6
H2A—C2—H2B	108.2	H6A—C6—H6B	108.1
C4—C3—C4^i^	109.48 (18)	C5^i^—C7—C5	109.57 (17)
C4—C3—C2	109.69 (12)	C5^i^—C7—H7A	109.8
C4^i^—C3—C2	109.69 (12)	C5—C7—H7A	109.8
C4—C3—H3	109.3	C5^i^—C7—H7B	109.8
C4^i^—C3—H3	109.3	C5—C7—H7B	109.8
C2—C3—H3	109.3	H7A—C7—H7B	108.2
C3—C4—C5	109.26 (14)	C1—C8—Br1	113.35 (15)
C3—C4—H4A	109.8	C1—C8—H8A	108.9
C5—C4—H4A	109.8	Br1—C8—H8A	108.9
C3—C4—H4B	109.8	C1—C8—H8B	108.9
C5—C4—H4B	109.8	Br1—C8—H8B	108.9
H4A—C4—H4B	108.3	H8A—C8—H8B	107.7

Symmetry codes: (i) *x*, −*y*, *z*.
